# Plasma levels of high-mobility group box 1 and soluble receptor for advanced glycation end products in primary antiphospholipid antibody syndrome patients

**DOI:** 10.1371/journal.pone.0178404

**Published:** 2017-05-30

**Authors:** Kuo-Tung Tang, Tsu-Yi Hsieh, Ya-Hsuan Chao, Meng-Xian Lin, Yi-Hsing Chen, Der-Yuan Chen, Chi-Chen Lin

**Affiliations:** 1 Division of Allergy, Immunology and Rheumatology, Taichung Veterans General Hospital, Taichung, R.O.C; 2 Ph.D. Program in Translational Medicine, National Chung Hsing University, Taichung, R.O.C; 3 Institute of Biomedical Science and Rong Hsing Research Center for Translational Medicine, National Chung-Hsing University, Taichung, R.O.C; 4 School of Medicine, National Yang-Ming University, Taipei, R.O.C; 5 Department of Medical Research, Taichung Veterans General Hospital, Taichung, R.O.C; 6 Institute of Microbiology and Immunology, Chung Shan Medical University, Taichung, R.O.C; Peking University First Hospital, CHINA

## Abstract

**Introduction:**

Many studies have demonstrated elevated circulating levels of high-mobility group box 1 (HMGB1) and decreased circulating levels of soluble receptor for advanced glycation end products (sRAGE) in patients with autoimmune diseases. In the present study, we investigated plasma levels of both HMGB1 and sRAGE in primary antiphospholipid syndrome (pAPS) patients.

**Methods:**

We prospectively recruited 11 pAPS patients, 17 antiphospholipid antibody (APA)-positive SLE patients without APS manifestations (APA^+^SLE) and 12 SLE patients with secondary APS (APS^+^SLE). We also recruited 10 healthy controls (HCs). Plasma levels of HMGB1 and sRAGE were determined using sandwich ELISA kits. In addition, plasma levels of HMGB1 were also determined using Western blot in 6 pAPS patients and 6 HCs.

**Results:**

There was no significant difference in plasma levels of HMGB1 measured by ELISA among subgroups of the enrolled subjects. In addition, there was no significant difference in plasma levels of HMGB1 measured by Western blot between pAPS patients and HCs. On the other hand, we observed a trend toward lower plasma levels of sRAGE in APA^+^SLE or APS^+^SLE patients when compared with HCs. However, there was no significant difference in plasma levels of sRAGE between pAPS patients and HCs, or between APA^+^SLE patients and APS^+^SLE patients.

**Conclusion:**

There was no significant difference in plasma levels of sRAGE or HMGB1 between pAPS patients and HCs. Plasma levels of sRAGE/HMGB1 could not be utilized to differentiate between APA^+^SLE and APS^+^SLE patients.

## Introduction

The high-mobility group box (HMGB) protein family consists of chromatin-binding proteins that modulate chromosomal structures and regulate transcription [1]. As a member of this protein family, HMGB1 is an endogenous danger signal released when immune cells are activated or cell death occurs [2]. Upon secretion, HMGB1 bind receptors such as toll-like receptors (TLRs) and the receptor for advanced glycation end products (RAGE) to promote an inflammatory response [3]. Soluble RAGE (sRAGE), a truncated form of RAGE, lacks the cytosolic and transmembrane domains and is composed of only the extracellular ligand-binding domain. Soluble RAGE has the same ligand-binding specificity as RAGE and acts as a ‘decoy’ by binding to pro-inflammatory ligands including HMGB1 [4]. In addition, Zong et al. demonstrated that RAGE forms homodimers at the plasma membrane, which leads to signal transduction. Soluble RAGE can bind to RAGE, and inhibit RAGE dimerization and subsequent activation of the nuclear factor κ-light-chain-enhancer of activated B cells (NF-κB) pathway [5].

Many studies have demonstrated elevated circulating levels of HMGB1 and decreased circulating levels of sRAGE in patients with autoimmune diseases such as rheumatoid arthritis (RA) [6,7], systemic lupus erythematosus (SLE) [8–10] and adult onset Still’s disease [11]. Therefore, activation of the RAGE axis may participate in the pathogenesis of autoimmunity. Antiphospholipid antibody syndrome (APS) is an autoimmune disease characterized by the presence of antiphospholipid antibodies (APA) and vascular thrombosis or obstetrical complications [12]. APA is frequently associated with SLE [12] and, interestingly, some individuals present with positive APA but remain thrombosis-free [13]. In addition, HMGB1 and sRAGE have been reported to be associated with vascular thrombosis [14,15]. We hypothesized that elevated circulating levels of HMGB1 and decreased circulating levels of sRAGE are also present in primary APS (pAPS) patients, and both HMGB1 and sRAGE have a role in the differentiation between APA-positive SLE patients with and without thrombotic events. However, there are no data concerning the role of the RAGE axis in APS pathogenesis.

In the present study, we investigated plasma levels of both HMGB1 and sRAGE in pAPS patients, APA-positive SLE patients without APS menifestations (APA^+^SLE patients) and SLE patients with secondary APS (APS^+^SLE patients). Our results demonstrated no difference in plasma levels of sRAGE or HMGB1 between pAPS patients and HCs.

## Materials and methods

### Patients

We prospectively recruited 40 patients including 11 pAPS patients, 17 APA^+^SLE patients and 12 APS^+^SLE patients. The diagnosis of APS was made based on the revised Sapporo classification criteria [16]. All patients with SLE fulfilled the 1997 American College of Rheumatology criteria [17] except for two patients who had biopsy-proven nephritis compatible with SLE and antinuclear antibody (ANA)/anti-double-stranded DNA (anti-dsDNA) antibodies [18]. Disease activity of SLE was determined by the Systemic Lupus Erythematosus Disease Activity Index (SLEDAI) and a disease flare was defined as SLEDAI ≧ 4 [19]. We also recruited 10 healthy controls (HCs) without chronic disorders such as autoimmune diseases, etc. The Institutional Review Board of Taichung Veterans General Hospital approved this study (IRB TCVGH NO: CF14256A) and written consent from all participants was obtained according to the Declaration of Helsinki.

### Determination of immunologic parameters

Immunologic parameters were determined as previously described [20]. Serum anti-dsDNA, anticardiolipin antibodies (ACA) and anti-β2-glycoprotein I antibodies were determined using enzyme-linked immunosorbent assay (ELISA) kits (INOVA Diagnostics, Inc., San Diego, CA, USA). Lupus anticoagulant was examined with a clot detection method (Beckman Coulter, Inc., Brea, CA, USA). Complement 3 (C3) and complement 4 (C4) were determined using PEG-enhanced immunoturbidimetry (Siemens Healthcare Diagnostics Inc, Tarrytown, NY, USA).

### Determination of plasma levels of HMGB1 by ELISA

Plasma levels of HMGB1 were determined using sandwich ELISA kits (Chondrex, Redmond, WA) in accordance with the manufacturer’s instructions. Briefly, one hundred microliters of the capture antibody were precoated in each well of the microtiter plates at 4°C overnight. Fifty microliters of patient plasma or protein standards were added to the wells. One hundred microliters of detection antibody was added to the wells and incubated for 1hr at 37°C. After five washes, 100μl of streptavidin peroxidase was added to the wells and incubated for 30 minutes at room temperature. Five additional washes were performed and detection was performed with tetramethylbenzidine solution. For each sample, measurements were repeated twice and an average value was obtained.

### Determination of plasma levels of HMGB1 by Western blot

Owing to the poor correlation between serum levels of HMGB1 measured by ELISA and Western blot in SLE patients [21], we also measured plasma levels of HMGB1 by Western blot in 6 pAPS patients and 6 HCs. First, 1ul of plasma sample was diluted with 24 ul RIPA buffer and heated at 95°C for 5 minutes in SDS-loading buffer. Proteins were separated by 12% SDS-PAGE in running buffer (25mM Tris, 192mM glycine, 0.1% SDS). The gel was run at 70V for 30 minutes then at 100V until the blue dye front reached the bottom. The gel was transferred to polyvinylidene difluoride membrane (PVDF) in transfer buffer (50 mM Tris, 384 mM glycine, 20% methanol) at 300mA 1hr with the Mini TBC Electrophoretic Transfer Cell (BIO-RAD., Hercules, CA, USA). The membranes were blocked with 5% skimmed milk in TBST (150 mM NaCl, 20 mM Tris–HCl [pH 7.4], 0.1% Tween-20) at room temperature for 1hr and then probed with anti-HMGB1 rabbit antibody (1:5000, Abcam, Cambridge, MA, USA) at 4°C overnight. The membranes were washed 3 times with TBST, followed by incubation with peroxidase-conjugated secondary goat anti-rabbit IgG antibody (1:10000, Millipore, Darmstadt, Germany) at room temperature for 1hr. The membranes were washed 3 times with TBST again. Blots were developed by ECL detection system (Advansta, Menlo Park, CA USA) and exposed to X-ray film.

### Determination of plasma levels of sRAGE

Plasma levels of sRAGE were determined using sandwich ELISA kits (Sunglong Biotech, HangZhou, China) in accordance with the manufacturer’s instructions. Briefly, the wells had been pre-coated with sRAGE monoclonal antibody. Forty microliters of patient plasma plus 10ul of biotinylated sRAGE antibody, or 40 ul of protein standards (already combined with biotinylated antibody) were added to the wells. Five washes were performed and detection was performed with using streptavidin-HRP. For each sample, measurements were repeated twice and an average value was obtained.

### Statistics

Statistical analysis was performed using SPSS software version 22.0 (SPSS, Inc., Chicago, IL, USA.). All quantitative data were presented in medians plus the interquartile range unless otherwise specified. Mann-Whitney U test was used for between-group comparison of plasma levels of HMGB1 and sRAGE. The relationships between subgroups of the enrolled subjects and log-transformed plasma levels of HMGB1 or sRAGE were further examined in a multivariate linear regression model. The correlations between plasma levels of HMGB1, plasma levels of sRAGE and ACA IgG/IgM titers among the enrolled patients were examined using non-parametric Spearman’s correlation test. The correlations between plasma levels of HMGB1, plasma levels of sRAGE, serological markers of SLE disease activity such as anti-dsDNA, C3 and C4, and SLEDAI among SLE patients were also examined using non-parametric Spearman’s correlation test. The results of the correlation analyses were confirmed in 1000 bootstrap samples, with resampling of both variables. A two-tailed p value less than 0.05 was considered statistically significant. Bonferroni’s correction was applied for multiple comparisons.

## Results

### Demographic data and clinical characteristics of the enrolled subjects

Baseline characteristics of the enrolled subjects are illustrated in Table 1. The median age was 46 (39–52), 38 (29–52), 39 (28–51) and 34 (30–36) years respectively in pAPS, APA^+^SLE, APS^+^SLE patients and HCs. Although Kruskal-Wallis test demonstrated no significant difference in age between subgroups of the enrolled subjects, pairwise comparisons showed that HCs were younger than pAPS patients (p<0.005). The enrolled patients received hydroxychloroquine, antiplatelet drugs (aspirin or clopidogrel), anticoagulants (warfarin or enoxaparin), corticosteroids, immunosuppressants (methotrexate, azathioprine, mycophenolate mofetil, cyclosporine or cyclophosphomide) and/or biologics (rituximab or belimumab). APA^+^SLE patients received higher doses of corticosteroids compared with pAPS patients (7.5mg vs. 0mg prednisone per day). Otherwise, there were no significant differences in the disease duration, or the proportion of hydroxychloroquine, antiplatelet drugs, anticoagulants, immunosuppressants or biologics used among subgroups of the enrolled patients, although there was a trend toward an increased proportion of warfarin used in pAPS patients when compared to that in APA^+^SLE patients (46% vs. 12%, p = 0.076). Four patients (one pAPS and 3 APA^+^SLE patients) did not receive a measurement of plasma level of sRAGE.

**Table 1:** Baseline characteristics of the enrolled subjects.

	pAPS patients (n = 11)	APA^+^SLE patients (n = 17)	APS^+^SLE patients (n = 12)	HCs (n = 10)
Age (years)	46 (39–52)	38 (29–52)	39 (28–51)	34 (30–36)
Proportion of female	91%	82%	92%	70%
Disease duration (years)	10 (4–13)	13 (9–17)	10 (7–18)	N.A.
Presence of APA				
Lupus anticoagulant	9 (82%)	15 (88%)	12 (100%)	N.A.
Anticardiolipin antibody IgG/M	5 (45%)	10 (59%)	5 (42%)	N.A.
Anti-B2GP1 antibody IgG/M	2 (18%)	5 (29%)	1 (8%)	N.A.
Manifestations of APS				
Thrombocytopenia	3 (27%)	0 (0%)	0 (0%)	N.A.
TIA/stroke	2 (18%)	0 (0%)	4 (33%)	N.A.
Spontaneous abortion	1 (9%)	0 (0%)	3 (25%)	N.A.
Peripheral arterial disease	1 (9%)	0 (0%)	1 (8%)	N.A.
Nephropathy	1 (9%)	0 (0%)	0 (0%)	N.A.
Venous thromboembolism	2 (18%)	0 (0%)	3 (25%)	N.A.
Central retinal venous occlusion	1 (9%)	0 (0%)	1 (8%)	N.A.
Livedo reticularis	0 (0%)	0 (0%)	1 (8%)	N.A.
Medication use				
Hydroxychloroquine	10 (91%)	14 (82%)	10 (83%)	N.A.
Aspirin	6 (55%)	11 (65%)	8 (67%)	N.A.
Warfarin	5 (46%)	2 (12%)	3 (25%)	N.A.
Corticosteroid (mg)*	0 (0–4.4)	7.5 (5.0–10.0)	5.0 (0.6–6.9)	N.A.
Biologics	1 (9%)	2 (12%)	0 (0%)	N.A.

*p<0.05, Kruskal-Wallis test

APA, antiphospholipid antibody; B2GP1, β2-glycoprotein I; HC, healthy control; HMGB1, high-mobility group box 1; N.A., not available; pAPS, primary antiphospholipid syndrome; SLE, systemic lupus erythematosus; sRAGE, soluble receptor for advanced glycation end products.

### Comparisons of plasma levels of HMGB1 measured by ELISA between subgroups of the enrolled subjects

**Fig 1:**
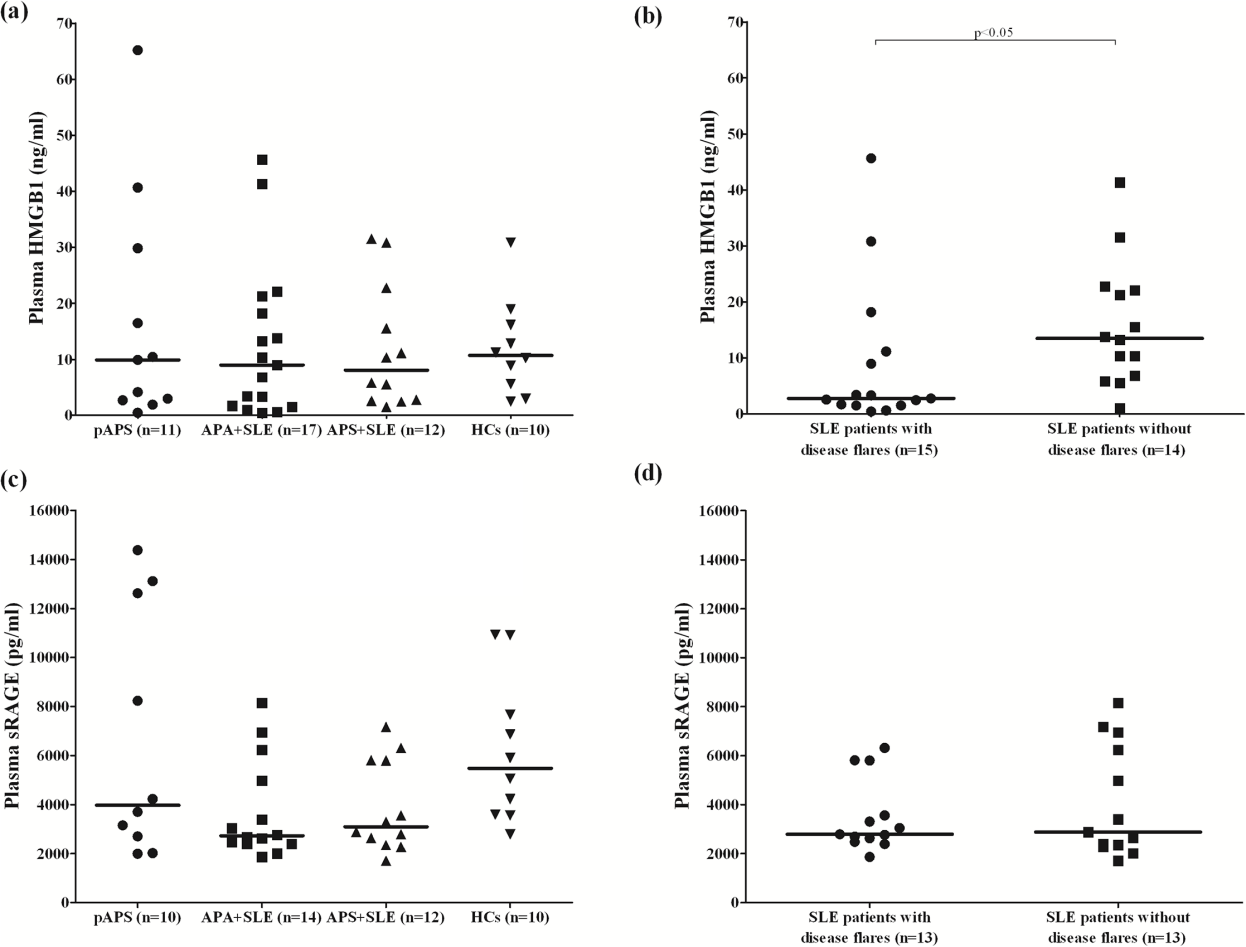
Comparisons of plasma levels of HMGB1 and sRAGE measured by ELISA between subgroups of the enrolled patients and HCs. (a) Comparison of plasma levels of HMGB1 between pAPS, APA^+^SLE, APS^+^SLE patients and HCs. (b) Comparison of plasma levels of HMGB1 between SLE patients with and without disease flares. (c) Comparison of plasma levels of sRAGE between pAPS, APA^+^SLE, APS^+^SLE patients and HCs. (d) Comparison of plasma levels of sRAGE between SLE patients with and without disease flares. P values of < 0.008 were regarded as statistically significant for multiple comparisons. APA, antiphospholipid antibody; HC, healthy control; HMGB1, high-mobility group box 1; pAPS, primary antiphospholipid syndrome; SLE, systemic lupus erythematosus; sRAGE, soluble receptor for advanced glycation end products.

As illustrated in Fig 1A, the median plasma levels of HMGB1 was 9.89 (2.85–23.15) ng/ml in pAPS patients, 8.99 (1.71–18.18) ng/ml in APA^+^SLE patients, 8.09 (2.68–19.17) ng/ml in APS^+^SLE patients, and 10.70 (5.58–16.15) ng/ml in HCs. There was no significant difference in plasma levels of HMGB1 among these groups of the enrolled subjects. Interestingly, SLE patients with disease flares had lower plasma levels of HMGB1 than those patients without disease flares (2.78 [1.51–11.15] ng/ml vs. 13.50 [6.59–22.27] ng/ml, p<0.05, Fig 1B).

### Comparisons of plasma levels of HMGB1 measured by Western blot between pAPS patients and HCs

**Fig 2:**
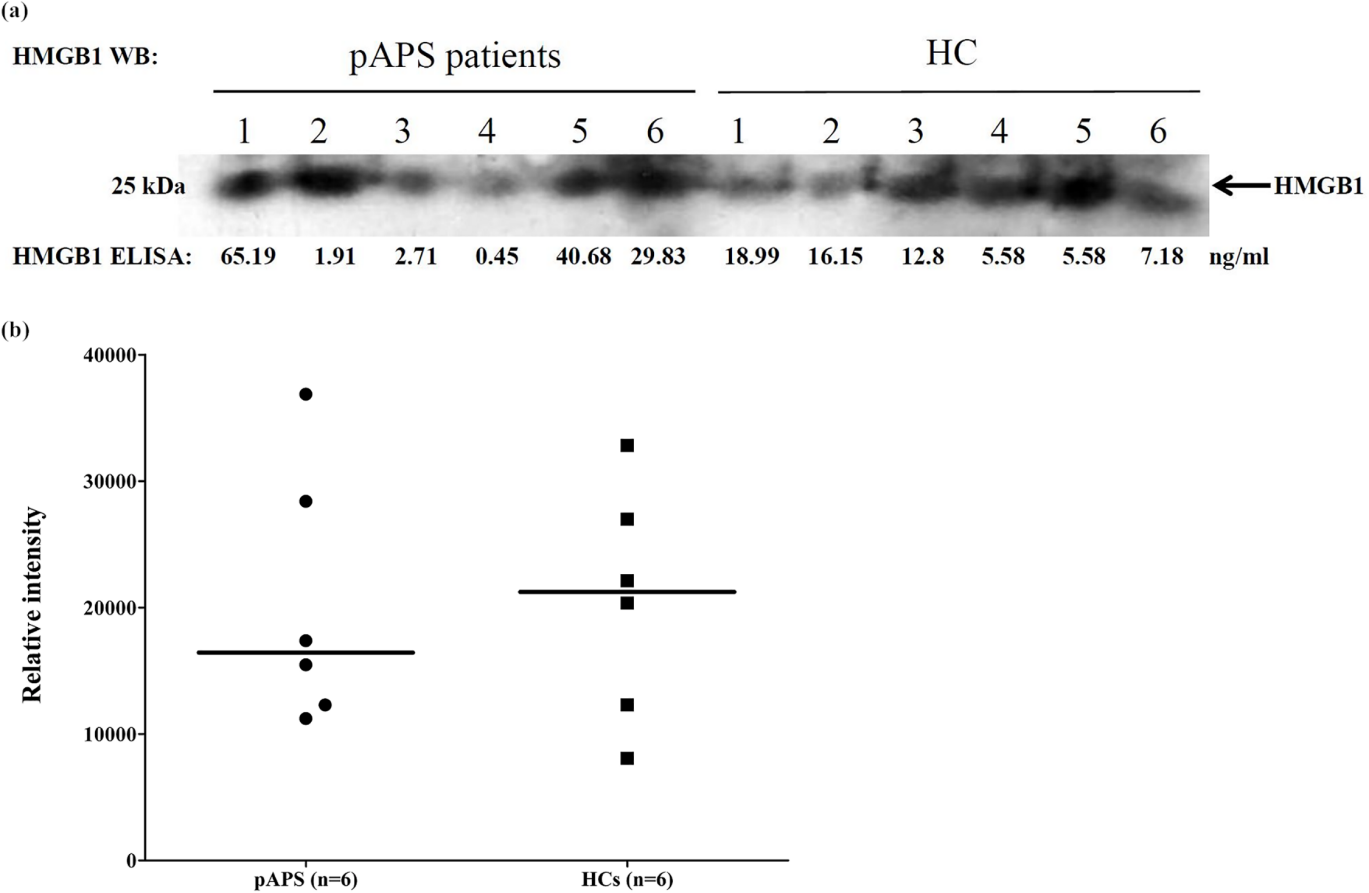
Comparisons of plasma levels of HMGB1 measured by Western blot between pAPS patients and HCs. (a) Poor correlation of ELISA with Western blot in the measurement of plasma levels of HMGB1 in both pAPS patients and HCs. (b) Comparison of plasma levels of HMGB1 between pAPS patients and HCs. Band intensities were quantified by ImageJ. HC, healthy control; HMGB1, high-mobility group box 1; pAPS, primary antiphospholipid syndrome; WB, Western blot.

As illustrated in Fig 2A, there was a poor correlation between levels of HMGB1 measured by ELISA and Western blot in both pAPS patients and HCs. There was no difference in plasma levels of HMGB1 measured by Western blot between pAPS patients and HCs (Fig 2B).

### Comparisons of plasma levels of sRAGE between subgroups of the enrolled subjects

As illustrated in Fig 1C, the median plasma levels of sRAGE was 3970 (2534–12748) pg/ml in pAPS patients, 2728 (2397–5285) pg/ml in APA^+^SLE patients, 3094 (2425–5810) pg/ml in APS^+^SLE patients, and 5486 (3586–8483) pg/ml in HCs. The plasma levels of sRAGE were lower in APA^+^SLE or APS^+^SLE patients compared with HCs (p = 0.012 and 0.029 respectively), although the statistical significance was not reached after Bonferroni’s correction. There was no significant difference in plasma levels of sRAGE between pAPS patients and HCs, or between APA^+^SLE patients and APS^+^SLE patients.

### The relationships between subgroups of the enrolled subjects and plasma levels of HMGB1/sRAGE in a linear regression model

Table 2 illustrates the relationships between subgroups of the enrolled subjects and log-transformed plasma levels of HMGB1/sRAGE in a linear regression model. After adjusting for age, gender and dosage of corticosteroids, APA^+^SLE and APS^+^SLE patients were associated with lower log-transformed plasma levels of sRAGE, but not HMGB1. In addition, dosage of corticosteroids was positively associated with log-transformed plasma levels of sRAGE.

**Table 2:** The relationships between subgroups of patients and log-transformed plasma levels of HMGB1 or sRAGE in a multivariate linear regression model (R^2^ = 0.04 for plasma HMGB1; R^2^ = 0.19 for plasma sRAGE).

	Log plasma HMGB1	Log plasma sRAGE
Parameters	β	β
Age	-0.34	0.16
Gender	-0.04	-0.17
Patient subgroups		
pAPS patients	0.02	-0.19
APA^+^SLE patients	0.01	-0.70**
APS^+^SLE patients	0.06	-0.58**
HCs	ref.	ref.
Dosage of corticosteroids	-0.19	0.38*

*P<0.05

**P<0.005.

ref.: reference

### Correlations between plasma levels of HMGB1, sRAGE and serum ACA IgG/IgM titers

**Fig 3:**
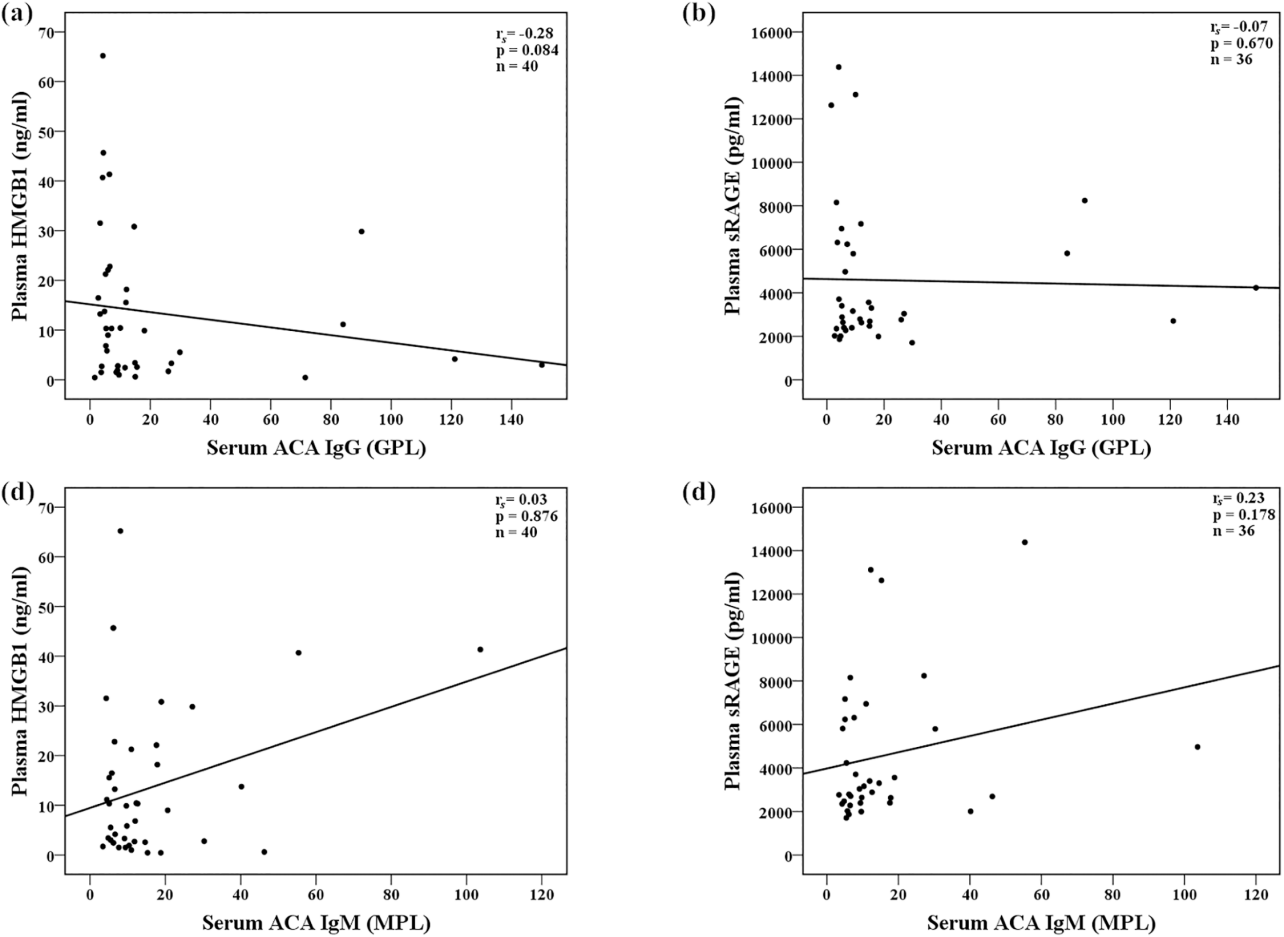
Correlation analyses between plasma levels of sRAGE, HMGB1 and serum ACA IgG or IgM titers among the enrolled patients. (a) Correlation between plasma levels of HMGB1 and serum ACA IgG titers; (b) Correlation between plasma levels of sRAGE and serum ACA IgG titers; (c) Correlation between plasma levels of HMGB1 and serum ACA IgM titers; (d) Correlation between plasma levels of sRAGE and serum ACA IgM titers. Correlation analyses were performed using Spearman’s rank correlation test. ACA: anticardiolipin antibodies; HMGB1, high-mobility group box 1; sRAGE, soluble receptor for advanced glycation end products.

As illustrated in Fig 3, there was no correlation between plasma levels of HMGB1/sRAGE and serum ACA IgG/IgM titers among the enrolled patients.

### Correlations between plasma levels of HMGB1, sRAGE, serological markers of disease activity and SLEDAI among enrolled SLE patients

**Fig 4:**
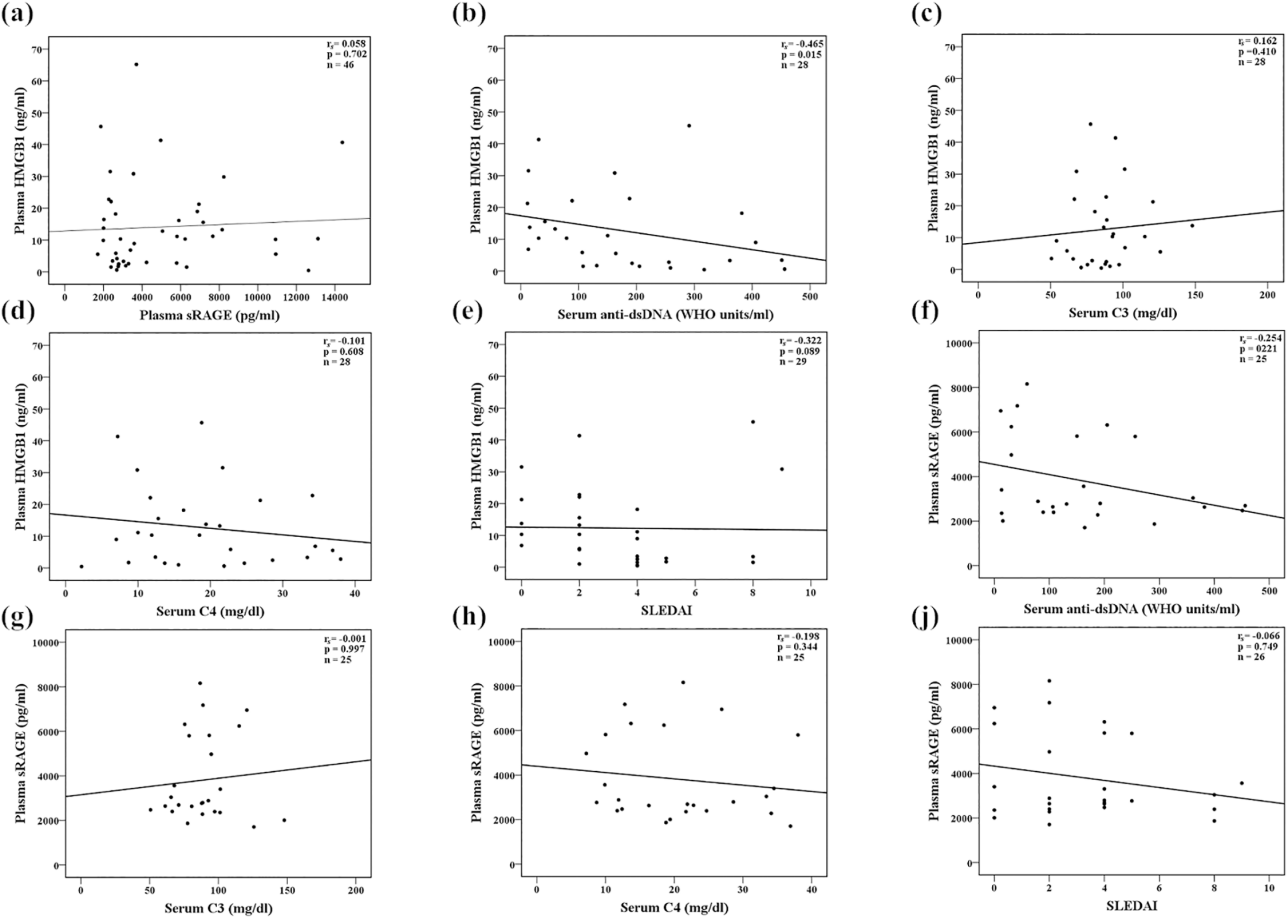
Correlation analyses between plasma levels of sRAGE, HMGB1, serological markers of disease activity and SLEDAI among SLE patients. (a) Correlation between plasma levels of HMGB1 and sRAGE; (b) Correlation between plasma levels of HMGB1 and serum anti-dsDNA; (c) Correlation between plasma levels of HMGB1 and serum C3; (d) Correlation between plasma levels of HMGB1 and serum C4; (e) Correlation between plasma levels of HMGB1 and SLEDAI; (f) Correlation between plasma levels of sRAGE and serum anti-dsDNA; (g) Correlation between plasma levels of sRAGE and serum C3; (h) Correlation between plasma levels of sRAGE and serum C4; (i) Correlation between plasma levels of sRAGE and SLEDAI. Correlation analyses were performed using Spearman’s rank correlation test. ^a^One APS^+^SLE patient did not receive anti-dsDNA, C3 and C4 examinations. APS, antiphospholipid syndrome; C3, complement 3; C4, complement 4; anti-dsDNA, anti-double-stranded DNA antibody; HMGB1, high-mobility group box 1; SLE, systemic lupus erythemtosus; SLEDAI, Systemic Lupus Erythematosus Disease Activity Index; sRAGE, soluble receptor for advanced glycation end products.

As illustrated in Fig 4, there was a weak inverse correlation between plasma levels of HMGB1 and anti-dsDNA (correlation coefficient: -0.465, p = 0.015). In addition, there was a trend toward an inverse correlation between plasma levels of HMGB1 and SLEDAI (correlation coefficient: -0.322, p = 0.089). Otherwise, there were no significant correlations between plasma levels of HMGB1, sRAGE, serological markers of SLE disease activity and SLEDAI.

## Discussion

This study is the first attempt to investigate the plasma levels of HMGB1 and sRAGE in pAPS patients. In contrast to previous reports regarding many autoimmune diseases such as RA and SLE, we did not find a significant difference in plasma levels of HMGB1 or sRAGE between pAPS patients and HCs. In addition, plasma levels of sRAGE or HMGB1 could not be utilized to differentiate between APA^+^SLE and APS^+^SLE patients.

Studies have demonstrated elevated circulating levels of HMGB1 and decreased circulating levels of sRAGE in autoimmune diseases [6–10]. Consistent with previous studies [9,10], we found a trend toward lower plasma levels of sRAGE in APA-positive SLE patients, with or without APS manifestations, when compared to HCs. However, we did not find a significant difference in plasma levels of HMGB1 between SLE patients and HCs. As implicated in our previous study [20], the interference of anti-HMGB1 antibodies in ELISA measurement might contribute to such findings. The presence of anti-HMGB1 antibodies also explained our results showing lower plasma levels of HMGB1 in SLE patients with disease flares and an inverse correlation between plasma levels of HMGB1 and serum anti-dsDNA in SLE patients [20,22].

In the present study, there was no difference in plasma levels of sRAGE between pAPS patients and HCs, and no correlations between plasma levels of sRAGE and serum ACA IgG or IgM titers. Thrombosis in APS is triggered by APA, which then stimulates endothelial cells and monocytes to express tissue factor and activate downstream coagulation cascade [12]. Therefore, inflammation contributes, only in part, to the pathogenesis of APS, as reflected by the poor efficacy of corticosteroids in APS patients [23] and by the main treatment for APS with anticoagulants [24]. In contrast, inflammation predominantly underlies the pathogenesis of autoimmune diseases such as RA and SLE [25,26]. Could this difference partly explain why we did not find decreased plasma levels of sRAGE in pAPS patients, as occurs in patients with other autoimmune diseases? It is difficult to answer based on our preliminary results. On the other hand, studies have demonstrated an association between atherosclerosis, a contributor to vascular thrombosis, and lower plasma levels of sRAGE [15,27]. In this regard, our contradictory findings suggested different pathogenic mechanisms between APS and atherosclerosis [28], as reflected by the conflicting evidence for an accelerated atherosclerosis in pAPS patients [29,30].

We also did not find a significant difference in plasma levels of HMGB1 (measured by either ELISA or Western blot) between pAPS patients and HCs, nor did we find correlations between plasma levels of HMGB1 and serum ACA IgG or IgM titers. As discussed above about plasma levels of sRAGE, there was no good explanation for such findings. Although not often examined in pAPS patients, some studies indicated that the production of autoantibodies is prevalent in pAPS patients [31,32]. Interestingly, we observed a poor correlation between plasma levels of HMGB1 measured by ELISA and Western blot. It is possible that the presence of anti-HMGB1 antibodies in pAPS patients interfered with the ELISA measurement of HMGB1 [20,21]. This speculation needs further confirmation with concomitant measurement of anti-HMGB1 antibodies in pAPS patients.

In clinical practice, it is controversial to prescribe hydroxychloroquine and/or aspirin as primary prophylaxis in patients with positive APA but without thrombosis events [33]. Previous studies have demonstrated that patients with high risk APA profiles, other cardiovascular risk factors or concomitant SLE are at increased risk for future thrombosis [34]. However, no biomarker can help in the prediction of future thrombosis risk in APA-positive patients. We did not find a significant difference in plasma levels of sRAGE or HMGB1 between APA^+^SLE and APS^+^SLE patients. Therefore, plasma levels of sRAGE or HMGB1 could not be utilized to differente between APA^+^SLE and APS^+^SLE patients.

There was no significant correlation between plasma levels of HMGB1 and sRAGE in our study, which is consistent with previous findings [10]. This implied that the RAGE axis involves not only HMGB1 but also some other ligands such as AGEs, the S100/calgranulin family and Mac-1 [35]. The interplay of these ligands, sRAGE and RAGE determines downstream inflammatory responses [10]. Interestingly, plasma levels of sRAGE were not correlated with SLE disease activity (serological markers or SLEDAI) in the present study. Therefore, plasma levels of sRAGE did not reflect lupus disease activity although they were lower in SLE patients when compared with HCs.

This finding was consistent with that of Ma et al., who found that plasma levels of sRAGE were comparable between untreated and treated SLE patients [9]. On the contrary, Yu et al. demonstrated that plasma levels of sRAGE were negatively correlated with SLEDAI in patients with lupus nephritis [10]. More studies are needed to provide a definite answer.

Our study has some limitations. First, the sample size is small, which reduces the statistical power of the study. Second, HCs were younger than pAPS patients and the median corticosteroid dosage was different between subgroups of the enrolled subjects.

However, our findings did not change after adjusting for these variables in a multivariate linear regression model. Third, patients in our study were not newly-diagnosed and already received medications. Therefore, we could not precisely evaluate the effect medications exert on plasma levels of HMGB1 or sRAGE. Fourth, four patients (one pAPS and 3 APA^+^SLE patients) did not receive a measurement of plasma level of sRAGE in the present study, which might bias our results. However, our findings in plasma levels of HMGB1 and sRAGE, both being components of the RAGE axis, were consistent. Fifth, most of our APS patients manifested as vascular thrombosis but not obstetric complications. Therefore, our results cannot be extrapolated to patients with obstetric APS, in view of its apparently different pathogenic mechanism from vascular APS [36].

In conclusion, there was no significant difference in plasma levels of sRAGE or HMGB1 between pAPS patients and HCs. Plasma levels of sRAGE or HMGB1 could not be utilized to differentiate between APA^+^SLE and APS^+^SLE patients.

## Supporting information

S1 File The original dataset of the study. (XLS)
